# Risk and protective factors for marijuana use and problems with use in a national sample of female Ecuadorian secondary school students

**DOI:** 10.1002/ijop.12881

**Published:** 2022-09-23

**Authors:** Daniela Ocaña‐Gordillo, Wendy Kliewer

**Affiliations:** ^1^ Parametría Consultancy Quito Ecuador; ^2^ Department of Psychology Virginia Commonwealth University USA

**Keywords:** Ecuador, Marijuana, Females, Risk factors, Protective factors

## Abstract

This study investigated risk and protective factors for marijuana use and problems with use in Ecuadorian girls in an attempt to inform this growing problem. Female secondary school students (*N* = 16,310; *M* = 15.02, *SD* = 1.73 years) who completed the 2016 national survey of drug use participated. The likelihood of lifetime marijuana use, reported by 7.3% of the sample, was predicted by older age, greater perceived access to marijuana and affiliation with tobacco‐ and marijuana‐using peers; aspects of parental monitoring and perceived physical safety in and around school were negatively related to the probability of use. Among girls reporting any marijuana use, age, frequency of past year use, ease of access and affiliation with marijuana‐using peers was positively associated with marijuana use problems. These data support the roles of both parents and communities in reducing marijuana use among Ecuadorian girls and highlight the important role of peer influence.

Consistent with worldwide and South American trends (United Nations Office on Drugs and Crime [UNODC], 2021), during the last 8 years in Ecuador, marijuana use among adolescent females has increased, while perceptions of harm have decreased (Organization of American States [OAS], 2019a, 2019b) Additionally, marijuana has become more available as the Ecuadorian government has worked to legalise medical marijuana use (Parametría Consultores, 2020).

Recent reviews highlight the consequences of youth marijuana use, supporting concerns about increases in usage. For example, in their review of high quality, longitudinal studies on this topic, Scheier and Griffin (2021) found consistent evidence that early initiation and ongoing use of marijuana was associated with impaired cognitive functioning, compromised health and increased risk of psychiatric problems. Furthermore, two of the most critical reasons to focus on females are that (a) risk and protective factors associated with marijuana use differ by sex (Kliewer et al., 2019; Wan et al., 2019) and (b) although more men than women develop substance use disorders (SUDs), when women do develop SUDs they evidence worse outcomes than men. Medical, psychiatric and functional problems that covary with SUDs often are more severe in women compared to men (McHugh et al., 2018; OAS, 2019a). In order to better protect girls from the risks associated with marijuana use, an improved understanding of risk and protective factors associated with use and problems with use in Ecuador is needed.

Greater perceived ease of access to substances and more peer substance use have been associated with more adolescent substance use, while higher levels of parental monitoring and higher levels of feeling physically safe at school or in the community have been associated with less adolescent substance use in both Latinx and non‐Latinx cultures (Edwards et al., 2019; Grigsby et al., 2014; Schulenberg et al., 2014). Thus, drawing on a nationally representative sample of Ecuadorian female secondary school students, the present study evaluated risk and protective factors associated with lifetime marijuana use and problems with use. To our knowledge, this is the first study focused on Ecuadorian female adolescents to report such analyses. Based on previous literature, we anticipated that greater perceived access to marijuana and more affiliation with substance‐using peers would be positively associated with the likelihood of marijuana use and would predict problems with use, while higher levels of parental monitoring and higher levels of feeling unsafe in and around school would be reduce the likelihood of marijuana use and problems with use.

## METHOD

### Participants and study design

All female Ecuadorian secondary school students who completed a national survey of drug use in 2016 and who had valid data on age and lifetime marijuana use were included in the study (*N* = 16,310). Girls ranged in age from 11 to 18 (*M* = 15.02, *SD* = 1.73) and most (59%) lived with both biological parents, with another 7.9% living with a mother or father and a stepparent.

### Procedure

The survey was approved by the Ministries of Health and of Education and was completed anonymously. Students consented for their data to be analysed and reported. The sampling plan was designed by the National Institute for Census and Statistics in Ecuador and involved two phases. In phase one, a random sample of public and private schools serving youth in grades equivalent to 9th, 11th and 13th in the United States were identified from the 24 capital cities of Ecuador's provinces, other urban areas in the country and cities with more than 30,000 inhabitants. In phase two, a random sample of grade 9, 11 and 13 classrooms in those schools were identified for assessment. University students, who were not connected to school staff, oversaw the survey administration, which took place during normal school hours. Students took between 20 and 60 minutes to complete the survey. No compensation was provided.

All procedures performed in this study were in accordance with the ethical standards of the Ecuadorian government and with the 1964 Helsinki Declaration and its later amendments or comparable ethical standards. Written consent from parents for Ecuadorian national school‐based surveys is not typically obtained and was not obtained for this survey.

### Measures

#### Marijuana use history and problems with use

Students were asked if they had ever consumed marijuana in their entire life (0 = *no*, 1 = *yes*) and how often they smoked marijuana in the last year. Responses for past year marijuana use ranged from 1 (*once a year*) to 5 (*5 to 7 days per week*). Problematic use was assessed with six questions reflecting varied behaviours and consequences associated with use, including smoking marijuana before noon, smoking alone, having memory problems when smoking, being told by family or friends that they should cut down on marijuana use, unsuccessful attempts to cut down on use and having problems such as fights, accidents, poor academic performance, so forth due to use of marijuana. This measure was used in prior surveys of Ecuadorian youth (National Council for the Control of Narcotic and Psychotropic Substances, 2008, 2012, 2015). The validation study is reported in Llorens (2013). Initial response options were 1 (*never*), 2 (*very rarely*), 3 (*occasionally*), 4 (*quite often*) and 5 (*very often*). Based on distributions, items were dichotomized by combining the *never* and *very rarely* responses (coded 0) and the *occasionally, quite often* and *very often* responses (coded 1). The number of items with responses of “1” were tallied, yielding a measure of marijuana use problems with a possible range from 0 to 6.

#### Risk and protective factors

Measures of risk and protective factors were used in prior studies of Ecuadorian youth and their validation is reported in the Inter‐American Observatory for Drugs (2011).

##### Risk factors

*Ease of access to marijuana* was assessed with one question “Would it be easy or difficult for you to get marijuana?” Responses ranged from 1 (*could not get marijuana*) to 3 (*it would be easy*); higher values reflected greater ease of access and more risk. *Peer substance use* was assessed with three questions reflecting the number of friends at school who used alcohol, tobacco and marijuana, respectively. Responses ranged from 1 (*all of them; 100%*) to 5 (*none of them; 0%*) and were recoded so that higher values reflected greater risk. Items were used separately in the analyses to isolate the contributions of specific substances.

##### Protective factors

*Parental monitoring* was assessed with six items reflecting parental knowledge and monitoring of adolescents' activities. Response options varied by question but were recoded so that higher values indicated parental behaviour that was more protective. See Table 1 for a list of the items. Items were used as separate indicators in the analyses. *Perceived physical safety in and around school* was assessed with three items indicating perceived safety in school, around school and on the way from school to home. Responses ranged from 1 (*very secure*) to 5 (*very insecure*). Items were recoded and combined to form a measure of perceived safety from physical harm. Cronbach's alpha was .77.

**TABLE 1:** Descriptive Information on Study Variables

Variables	M	SD	Possible range
Ease of access to marijuana	1.47	0.78	1–3
Friends' alcohol use	2.03	1.15	1–5
Friends' tobacco use	1.69	1.03	1–5
Friends' marijuana use	1.58	0.99	1–5
Parents' knowledge of whereabouts	2.78	0.47	1–3
Parents' concern about school activities	3.35	0.74	1–4
Parents' rules about weekend curfew^a^	0.88	0.33	0–1
Parents' knowledge of closest friends	2.98	0.82	1–4
Parents' expectations regarding reporting activities	2.79	0.48	1–3
Parents' knowledge of internet use^a^	0.66	0.47	0–1
Physical safety in and near school	3.41	0.90	1–5
Age	15.02	1.73	11–18
Lifetime marijuana use^a^	0.073	—	0–1
Past year marijuana use^b^	2.45	1.51	1–5
Problematic marijuana use^b^	1.55	1.42	0–6

*Note*: Higher values indicate greater levels of each construct.

^a^ For these items, yes was coded 1 and no was coded 0.

^b^ Reported for lifetime users of marijuana only.

#### Demographics

Students answered questions on their biological sex, age and with whom they lived. Students marked everyone with whom they lived from comprehensive list of family members.

### Data analysis

SPSS version 27 was used to analyse the data. Binary logistic regression analysis was used to evaluate associations of risk and protective factors with the likelihood of ever using marijuana. For girls who ever reported using marijuana, linear regression was used to predict problematic use. Missing data was imputed using the multiple imputation command in SPSS.

## RESULTS

### Descriptive analyses

Overall, 7.3% of the sample reported any use of marijuana during their lifetime, with usage increasing by age from 1.6% of girls 12 and younger to 11.3% for girls 17 and older reporting lifetime use. Table 1 presents descriptive information on the study variables.

### Logistic regression analysis predicting the probability of lifetime marijuana use

Binary logistic regression was used to evaluate the unique contributions of age and risk and protective factors to the probability of lifetime marijuana use (see Table 2, left columns). All variables were entered simultaneously. Older age, greater perceived access to marijuana and friend's tobacco and marijuana use predicted the probability of lifetime marijuana use. Conversely, parental knowledge of adolescent whereabouts after school and on the weekends, of adolescents' friends and of internet use and greater perceptions of physical safety in and around school reduced the likelihood lifetime marijuana use.

**TABLE 2:** Regression predicting the likelihood of lifetime and problematic marijuana use from risk and protective factors

	Binary logistic regression predicting the likelihood lifetime marijuana use (N = 16,310)			Linear regression predicting marijuana use problems among lifetime marijuana users only (N = 1,190)			
Variable	OR	p	95% CI for OR	B	SE	p	95% CI B
Age	**1.28**	**<.001**	**[1.23, 1.34]**	**−.10**	**.03**	**<.001**	**[−.15, −.04]**
Past year frequency of marijuana use	—	**—**	—	**.27**	**.03**	**<.001**	**[.21, .33]**
Ease of perceived access to marijuana	**2.33**	**<.001**	**[2.16, 2.51]**	**.12**	**.05**	**.008**	**[.03, .21]**
Friends' use of alcohol	.98	.65	[.91, 1.06]	−.04	.04	.40	[−.12, .05]
Friends' use of tobacco	**1.11**	**.009**	**[1.03, 1.21]**	.02	.04	.67	[−.07, .10]
Friends' use of marijuana	**1.63**	**<.001**	**[1.52, 1.75]**	.15	**.04**	**<.001**	**[.08, .23]**
Parental knowledge of whereabouts after school and during the weekends	**.63**	**<.001**	**[.56, .71]**	.003	.07	.96	[−.13, .14]
Parental concern about school activities	**.91**	**.04**	**[.83, .99]**	−.05	.05	.40	[−.14, .26]
Parental rules about weekend curfew	.85	.08	[.71, 1.02]	.06	.10	.57	[−.09, .12]
Parental knowledge of closest friends	**.90**	**.03**	**[.82, .99]**	.01	.05	.79	[−.16, .10]
Parental expectations regarding reporting location of activities	**.84**	**.01**	**[.74, .96]**	−.08	.08	.29	[−.23, .07]
Parental knowledge of internet use	**.83**	**.01**	**[.72, .96]**	−.09	.08	.28	[−.26, .07]
Physical safety in and around school	.93	.20	[.84, 1.04]	.001	.06	.99	[−.12, .13]

*Note*: CI = confidence interval; OR = odds ratio. For both the logistic and linear regressions, all predictors were entered simultaneously. Significant associations are bolded. Regression results with imputed data are reflected in the table. In the logistic regression, 92.9% of the cases were correctly classified. Overall fit statistics for the linear regression: Adjusted *R* ^2^ = .11; *F*(13, 1176) = 12.28, *p* < .001.

### Linear regression analysis predicting problems with marijuana use

Table 2 (right columns) presents results of the regression analysis predicting problems with marijuana use for girls who reported ever using marijuana. In this model, frequency of past year marijuana use was included as a predictor. As seen in the table, the model was significant, explaining 11% of the variation in problematic marijuana use. Older age, frequent past year marijuana use, greater perceived access to substances and affiliation with marijuana‐using peers predicted more marijuana use problems.

## DISCUSSION

Two key results emerged from this nationally representative sample of Ecuadorian female adolescents. The first finding was that the probability of lifetime use of marijuana was associated with factors in different domains of adolescents' lives—family, peer group, school and community—with each playing a role in influencing marijuana use. Parental knowledge of adolescents' activities, friends' substance use, physical safety at school and access to drugs each were associated with lifetime use of marijuana while accounting for the contributions of other variables. These data are consistent with other studies with Latinx samples (e.g., Edwards et al., 2019; Grigsby et al., 2014) and with the broader literature on correlates of adolescent substance use (Schulenberg et al., 2014).

A second key result from the study was that once girls began to use marijuana, risk factors predominated. Perceived ease of access to substances and affiliation with marijuana‐using peers were key contributions to the likelihood of reporting problems associated with use. This data is consistent with research from Latin countries, including countries that border Ecuador (OAS, 2019a) and reflects the salience of peer influence, relative to family influence, during the period of early and middle adolescence. This finding is consistent with Degenhart et al. (2016) who found that relative to other risk factors, peer substance use was the strongest predictor of adolescent substance use.

There were a number of strengths to this study including the large, nationally representative sample, the focus on girls, inclusion of predictors from individual, family, peer and school domains and assessment of both lifetime use and problematic use of marijuana. However, the study also had limitations including the cross‐sectional design, which precluded determining the temporal order of associations. Additionally, we only were able to assess the influence of the risk and protective factors included in the survey. This meant that some key risk factors, such as trauma exposure and some key protective factors, such as emotion regulation, were not evaluated.

The study results suggest several directions for future research and practice. First, the fact that aspects of parental monitoring were significant for lifetime prevalence of marihuana use but not for problematic use among females suggests that early intervention that targets females and families before substance use exposure may be able to prevent marijuana misuse. Second, perceived access to marijuana was a predictor of both the likelihood of ever using and problematic use. This suggests that community involvement to reduce access could be an important preventive strategy. Third, the influence of peers that emerged in our data is consistent with studies that suggest that first time use of substances in females is correlated with use by a significant other who has a history of drug use (UNODC, 2016). This suggests that prevention efforts need to address all of the social relationships with which adolescent females are involved, including both peers and romantic partners. This suggestion applies to primary and secondary prevention efforts, as once a girl had tried marijuana, peer influences predominated in predicting problematic use.

In conclusion, results from this nationally representative sample of female Ecuadorian adolescents indicate that primary prevention strategies similar to those used around the globe—improving physical safety in and around schools, reducing access to marijuana, assisting parents in monitoring their children more effectively and addressing a peer culture that promotes substance use—may be effective in this context in reducing marijuana use. Furthermore, once use has begun, secondary prevention efforts should focus on each of the social relationships in which girls are involved.
